# Evaluation of Intralesional Triamcinolone Acetonide and Platelet‐Rich Plasma Therapy in Patients With Mild Patch‐Type Alopecia Areata

**DOI:** 10.1111/jocd.71120

**Published:** 2026-08-02

**Authors:** Ramazan Ülker, İlknur Yorğun Özdemir, Hatice Uce Özkol

**Affiliations:** ^1^ Department of Dermatology and Venereology Silopi Devlet Hastanesi Şırnak Türkiye; ^2^ Department of Dermatology, Faculty of Medicine Yüzüncü Yıl University Van Türkiye

**Keywords:** alopecia areata, intralesional corticosteroids, platelet‐rich plasma, SALT score, triamcinolone acetonide

## Abstract

**Introduction:**

Alopecia areata is a long‐term autoimmune disease that causes hair loss in approximately 2% of people but does not result in permanent scarring. Intralesional corticosteroid treatment provides effective results; however, long‐term use may lead to skin atrophy and telangiectasia. Platelet‐rich plasma (PRP), an autologous blood‐derived preparation, represents a patient‐specific alternative that avoids steroid‐related side effects.

**Objectives:**

To compare the efficacy and safety of intralesional PRP and intralesional triamcinolone acetonide (ILTA) in patients with mild patch‐type (S1) alopecia areata.

**Methods:**

This prospective study included patients aged 18–65 years with mild patch‐type alopecia areata who were admitted to our clinic between March 2024 and March 2025 and had not received any previous treatment. Patients were allocated into two groups; both groups received a total of three sessions of treatment every 4 weeks. The primary endpoint was the change in SALT score from baseline to the 6th month. Secondary endpoints included the change in SALT score at the 3rd month, clinical response categories, hair pull test conversion, relapse rate during 6‐month follow‐up, and the adverse‐event profile. SALT scores were calculated at baseline, 3 months, and 6 months.

**Results:**

Sixty patients (41 males, 19 females; mean age 27.97 ± 7.36 years) completed the study. SALT scores decreased significantly in both groups (*p* < 0.001). The score decreased from 6.03 to 0.93 in the PRP group and from 6.07 to 1.27 in the ILTA group, with no statistically significant difference between groups (mean difference 0.30; 95% CI −0.77 to 1.37; *p* = 0.576). The very good response rate (≥ 75% improvement) at six months was 76.67% in the PRP group and 66.67% in the ILTA group. Relapse occurred in 3 of 60 patients (5.0%) during the 6‐month follow‐up (1/30 (3.33%) in the PRP group and 2/30 (6.67%) in the ILTA group). Both groups experienced only mild adverse events; no serious side effects were observed.

**Conclusions:**

In mild untreated patch‐type alopecia areata, PRP showed similar efficacy to intralesional triamcinolone acetonide and may be considered an alternative treatment option for mild patch‐type alopecia areata.

## 1 Introduction

Alopecia areata (AA) is a non‐scarring autoimmune disease characterized by well‐demarcated, round patches of hair loss, and it affects people of all ages [1]. The exact cause of this chronic condition remains unknown, but genetic factors and immune system dysregulation are thought to play a central role in its development [2, 3]. The most common pathophysiological hypothesis is the disruption of the immune privilege of the hair follicle [4]. The point prevalence of alopecia areata is estimated at 0.1%–0.2% of the population, while the lifetime risk of developing the condition is approximately 2.1% [5]. A positive family history is reported in about 20% of patients, supporting the role of genetic predisposition. AA patients experience higher rates of anxiety and depression than the general population, as hair loss causes considerable psychological distress that affects their quality of life [6].

The diagnosis of this condition depends on standard clinical signs which trichoscopy and histopathology tests help confirm [7, 8]. Topical and intralesional steroids, minoxidil, calcineurin inhibitors, systemic corticosteroids, immunotherapies, anthralin, phototherapy, methotrexate, cyclosporine, JAK inhibitors, and platelet‐rich plasma (PRP) are used in the treatment [2, 4, 9]. However, long‐term use of intralesional triamcinolone acetonide (ILTA) may lead to side effects such as skin atrophy, telangiectasia, and hypopigmentation [10]. Assessing treatment efficacy is challenging because of the unpredictable course of the disease and the possibility of spontaneous remission [11].

PRP has been increasingly used in the treatment of AA in recent years. It was first applied in the 1970s as an autologous blood‐derived preparation for the management of thrombocytopenia [12]. The main advantage of PRP is its low risk of allergic reactions and systemic side effects due to its autologous nature [13]. For preparation, venous blood taken from the patient is centrifuged to obtain platelet‐rich plasma and injected into the target area [14].

The alpha granules of platelets release growth factors which include PDGF, TGF‐β, FGF‐2, VEGF, EGF, IGF‐1 and GDNF. Together, these factors promote hair growth by stimulating cell proliferation, cell differentiation, and angiogenesis [8, 12, 14]. GDNF protects the hair follicle from early catagen, while VEGF is involved in follicle stimulation by angiogenesis induction [12, 14]. PRP promotes the proliferation of dermal papilla cells through activation of the ERK, FGF‐7, β‐catenin, and Akt signaling pathways, and it reduces inflammation by lowering MCP‐1 levels [14]. Previous studies have reported that PRP achieves results comparable to those of intralesional corticosteroid injections [13]. Because its safety profile is independent of steroid‐related side effects, PRP is an appealing option for patients who require prolonged treatment.

In the present study, we compared intralesional PRP with ILTA in patients with patch‐type AA who were treated at our outpatient clinic.

## 2 Material and Methods

### 2.1 Study Population and Sample

This prospective controlled comparative study was conducted between March 2024 and March 2025 at our Dermatology Outpatient Clinic. The research included patients between 18 and 65 years old who had patchy alopecia areata and no history of previous medical treatment. The study excluded patients who had thrombocytopenia and hypofibrinogenemia, and those who experienced acute or chronic infections or autoimmune diseases, as well as patients who received anticoagulant therapy with warfarin, dabigatran, or heparin. The researchers documented two patch types, which they labeled as single or multiple, and they documented family history as either positive or negative.

The sample size was calculated a priori using G*Power version 3.1.9.7 (Heinrich Heine University Düsseldorf, Germany). The calculation was based on the expected between‐group difference in SALT score change at the 6‐month follow‐up, using the standard deviation of the SALT score change [8]. Assuming an expected between‐group mean difference of 1.5 SALT points, a common standard deviation of 2.0 SALT points (corresponding to a standardized effect size, Cohen's *d*, of approximately 0.76), a two‐sided significance level (*α*) of 0.05, a statistical power (1 − *β*) of 0.80, and an allocation ratio of 1:1, the required minimum sample size was 27 patients per group. The expected difference was defined in terms of the absolute change in SALT score rather than percentage improvement. A difference of this magnitude was considered clinically relevant in this cohort of mild, patch‐type (S1) disease, in which baseline SALT scores were low (approximately 6 points), since even a small absolute change represents a substantial proportional reduction in scalp involvement. To compensate for a potential dropout rate of approximately 10%, the final sample size was set at 30 patients per group, resulting in a total of 60 participants.

### 2.2 Operational Procedures

The clinical and demographic data were recorded prospectively; demographic characteristics, clinical findings, and hair pull test results were documented at each visit. The researchers used standardized clinical photographs to determine SALT scores at baseline and at 3 and 6 months. The SALT system organizes the scalp into four zones, comprising the vertex (40%), the right profile (18%), the left profile (18%), and the back (24%). The total score is obtained by multiplying the percentage of hair loss in each area by the relevant coefficient. The hair pull test was performed at each visit, and more than 6 hairs (more than 10%) extracted with gentle traction were considered positive. The patients were followed for 6 months, with evaluations at baseline, 3 months, and 6 months.

### 2.3 Outcomes

The primary outcome was the change in total SALT score from baseline to the 6th month. The secondary outcomes were: (1) the change in total SALT score from baseline to the 3rd month; (2) the distribution of clinical response categories at the 3rd and 6th months, defined as non‐response (< 25% reduction in SALT), partial response (25%–49%), good response (50%–74%), and very good response (≥ 75%); (3) the conversion of the hair pull test from positive to negative between baseline, the 3rd, and the 6th months; (4) the relapse rate during the 6‐month follow‐up period—relapse was operationally defined as the appearance of a new alopecic patch, or an increase of ≥ 25% in the SALT score compared with the post‐treatment nadir (3rd‐ or 6th‐month value), in a patient who had previously achieved at least a partial response (≥ 25% SALT reduction); and (5) the frequency and severity of treatment‐related adverse events (e.g., pain, burning, ecchymosis, erythema/edema, skin atrophy, telangiectasia, hypopigmentation). Adverse events were graded according to the Common Terminology Criteria for Adverse Events (CTCAE) version 5.0 [15].

### 2.4 Treatment (Intervention) Protocol

In the PRP group, venous blood was drawn into two 8 mL vacuum tubes and centrifuged in a Hettich EBA 200 centrifuge (Andreas Hettich GmbH, Tuttlingen, Germany) at approximately 1538 *g* (4000 rpm) for 4 min using a single‐spin protocol. A CE Class IIb–certified commercial kit (T‐LAB PRP Kit, T‐LAB Medical, Türkiye) was used. The platelet‐rich plasma layer was collected with an 18‐gauge needle, and 2–3 mL of PRP was obtained in each session. The platelet concentration was approximately 5–7 times the baseline serum value; this concentration corresponds to the validated yield of the kit and was not measured individually for each patient. No exogenous activator was added; activation was deliberately avoided so that gradual, physiological activation by dermal collagen would occur after injection, allowing a more sustained release of growth factors [16]. PRP was injected into the alopecia patches with a 30‐gauge needle at a dose of 0.05–0.1 mL/cm^2^, corresponding to a total of 2–3 mL per session distributed across the treated patches. Patients were advised not to use NSAIDs for 7 days before and after the procedure. In the ILTA group, triamcinolone acetonide (Kenacort‐A 40 mg/mL, Deva Holding, Türkiye) was diluted with normal saline to a concentration of 5 mg/mL. The solution was injected into the lower dermis with a 30‐gauge, 12–13 mm needle at 0.5–1 cm intervals. In both groups, treatment was administered every 4 weeks for a total of three sessions. Written informed consent was obtained from all patients.

### 2.5 Statistical Analysis

Statistical analyses were performed using SPSS version 21 (IBM Corp., Armonk, NY, USA). The Shapiro–Wilk test was used to assess the normality of continuous variables, and Levene's test was used to assess homogeneity of variances. Continuous variables were presented as mean ± standard deviation (SD), and categorical variables as numbers and percentages. Between‐group comparisons of normally distributed continuous variables were performed using the independent‐samples *t*‐test, while non‐normally distributed variables were compared using the Mann–Whitney *U* test. For the primary outcome, the between‐group difference in SALT score change was reported as the mean difference with its 95% confidence interval, and the effect size was calculated as Cohen's *d* using the pooled standard deviation. In addition, an analysis of covariance (ANCOVA) adjusting for the baseline SALT score was performed as a sensitivity analysis to confirm the robustness of the between‐group comparison [17]. Within‐group changes in SALT scores across the three time points (baseline, 3rd month, 6th month) were analyzed using repeated‐measures ANOVA for normally distributed data, or the Friedman test when the assumption of normality was not met; post hoc comparisons were adjusted with the Bonferroni correction. Where both parametric and non‐parametric approaches were applicable, the results were concordant. The relationship between categorical variables and treatment groups was analyzed using the chi‐square test or Fisher's exact test where appropriate. The effect of prognostic factors (gender, family history) on SALT score changes was evaluated using the independent‐samples *t*‐test. All tests were two‐tailed, and a *p*‐value of < 0.05 was considered statistically significant. All analyses were conducted on an intention‐to‐treat (ITT) basis; no missing data imputation was required, as all 60 patients completed the 6‐month follow‐up.

### 2.6 Ethical Considerations

Ethical approval was obtained from the Clinical Research Ethics Committee of Van Yüzüncü Yıl University (Approval No: 11063 Date: 28.01.2024) prior to the initiation of the study. The research team protected participant confidentiality through anonymous data collection and coding procedures, which also followed strict security protocols for encrypted systems. The study was conducted in accordance with the principles of the Declaration of Helsinki and Good Clinical Practice (GCP) guidelines.

## 3 Results

### 3.1 Patient Characteristics and Demographic Data

Of the 60 patients included in the study, 30 were in the PRP group and 30 were in the intralesional triamcinolone acetonide (ILTA) group. There were 20 males and 10 females in the PRP group and 21 males and 9 females in the ILTA group. According to the patch type, a single patch was detected in 23 patients and multiple patches in 37 patients. Eighteen patients had a positive family history. All patients had mild, S1‐type disease at baseline, and the two groups were comparable with respect to age, disease duration, sex, patch type, family history, and baseline SALT score (all *p* > 0.05) (Table 1).

**TABLE 1:** Demographic and clinical characteristics of the study population.

Variable	Category	PRP (*n* = 30)	ILTA (*n* = 30)	Overall (*n* = 60)	*p*
Age, years	—	28.20 ± 7.45	27.74 ± 7.30	27.97 ± 7.36	0.810
Disease duration, months	—	4.83 ± 7.05	5.17 ± 7.21	5.00 ± 7.11	0.854
Baseline SALT score	—	6.03 ± 0.89	6.07 ± 0.78	6.05 ± 0.83	0.854
Sex, *n* (%)	Male	20 (66.67)	21 (70.00)	41 (68.33)	0.781
	Female	10 (33.33)	9 (30.00)	19 (31.67)	
Patch type, *n* (%)	Single	12 (40.00)	11 (36.67)	23 (38.33)	0.791
	Multiple	18 (60.00)	19 (63.33)	37 (61.67)	
Family history, *n* (%)	Positive	9 (30.00)	9 (30.00)	18 (30.00)	1.000
	Negative	21 (70.00)	21 (70.00)	42 (70.00)	
SALT severity, *n* (%)	S1	30 (100.00)	30 (100.00)	60 (100.00)	—

*Note:* Continuous variables: mean ± SD; categorical variables: *n* (%). Continuous variables compared with the independent‐samples *t*‐test, categorical variables with the chi‐square test. *p* < 0.05 was significant.

Abbreviations: ILTA, intralesional triamcinolone acetonide; PRP, platelet‐rich plasma; S1, less than 25% scalp involvement; SALT, Severity of Alopecia Tool; SD, standard deviation.

### 3.2 Assessing Treatment Responses

In the PRP group, the baseline SALT score decreased from 6.03 to 2.53 in the third month and to 0.93 in the sixth month. In the ILTA group, the baseline value decreased from 6.07 to 2.60 at the third month and 1.27 at the sixth month. The improvement over time was statistically significant in both groups (repeated‐measures ANOVA, *p* < 0.001 within each group) (Table 2).

**TABLE 2:** Effect of prognostic factors on SALT score change.

Factor	Category	*n*	Baseline–3rd month	*p*	Baseline–6th month	*p*	3rd–6th month	*p*
Sex	Male	41	3.37 ± 2.07	0.951	5.00 ± 2.01	0.576	1.63 ± 1.95	0.568
	Female	19	3.74 ± 2.13		4.84 ± 2.19		1.11 ± 1.37	
Family history	Negative	42	3.38 ± 2.11	0.565	4.95 ± 2.04	0.989	1.57 ± 1.78	0.493
	Positive	18	3.72 ± 2.05		4.94 ± 2.15		1.22 ± 1.83	

*Note:* Values are mean ± SD of SALT score change between the indicated time points. Groups compared with the independent‐samples *t*‐test; *p* < 0.05 was significant.

Abbreviations: SALT, Severity of Alopecia Tool; SD, standard deviation.

**FIGURE 1:**
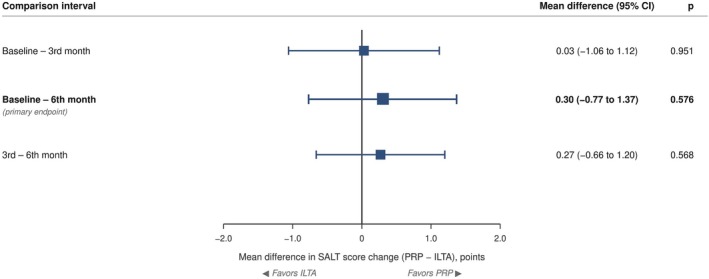
Between‐group differences in SALT score change between platelet‐rich plasma (PRP) and intralesional triamcinolone acetonide (ILTA). Forest plot showing the mean between‐group difference in SALT score change (PRP − ILTA) at three comparison intervals: Baseline to 3rd month, baseline to 6th month (primary endpoint), and 3rd to 6th month. Squares represent point estimates, scaled to the inverse variance, and horizontal lines indicate 95% confidence intervals. The vertical line at zero denotes no between‐group difference; values to the right favor PRP and values to the left favor ILTA. The confidence interval crosses the line of no effect at all three intervals, indicating no statistically significant between‐group difference. PRP, platelet‐rich plasma; ILTA, intralesional triamcinolone acetonide; SALT, Severity of Alopecia Tool; CI, confidence interval.

In the comparison between the groups, the baseline–third month SALT change was 3.50 in PRP and 3.47 in ILTA; the baseline–sixth month change was 5.10 for PRP and 4.80 for ILTA. For the primary endpoint, the mean between‐group difference in SALT change from baseline to the sixth month was 0.30 (95% CI −0.77 to 1.37; Cohen's *d* = 0.15; *p* = 0.576), and the difference at the third month was 0.03 (95% CI −1.06 to 1.12; *p* = 0.951). The confidence interval crossed zero at every time point, so no statistically significant between‐group difference was observed and the two treatments produced comparable improvement (Table 3, Figure 1).

**TABLE 3:** Within‐group and between‐group comparison of SALT scores.

Analysis	Period	PRP (*n* = 30)	ILTA (*n* = 30)	Difference (PRP–ILTA)	95% CI	Cohen's *d*	*p*
Within‐group	Baseline	6.03 ± 0.89	6.07 ± 0.78	−0.04	−0.47 to 0.39	0.05	0.854
	3rd month	2.53 ± 2.01	2.60 ± 2.08	−0.07	−1.13 to 0.99	0.03	0.898
	6th month	0.93 ± 1.72	1.27 ± 2.03	−0.34	−1.31 to 0.63	0.18	0.490
	Time effect	—	—	—	—	—	< 0.001^a^
Between‐group change	Baseline–3rd month	3.50 ± 1.89	3.47 ± 2.29	0.03	−1.06 to 1.12	0.01	0.951
	Baseline–6th month^b^	5.10 ± 1.90	4.80 ± 2.22	0.30	−0.77 to 1.37	0.15	0.576
	3rd–6th month	1.60 ± 1.98	1.33 ± 1.61	0.27	−0.66 to 1.20	0.15	0.568

*Note:* Values are mean ± SD.

Abbreviations: ANCOVA, analysis of covariance; ANOVA, analysis of variance; CI, confidence interval; ILTA, intralesional triamcinolone acetonide; PRP, platelet‐rich plasma; SALT, Severity of Alopecia Tool; SD, standard deviation.

^a^ Time effect across baseline, 3rd and 6th months within each group (repeated‐measures ANOVA, Bonferroni post hoc).

^b^ Primary endpoint. Between‐group differences tested with the independent‐samples *t*‐test after confirming normality (Shapiro–Wilk) and variance homogeneity (Levene); a baseline‐adjusted ANCOVA gave concordant results. Cohen's *d* calculated with the pooled SD. *p* < 0.05 was significant.

### 3.3 Impact of Prognostic Factors

The baseline–third month SALT change was 3.37 points for men and 3.74 points for women; the baseline–sixth month change was 5.00 points for men and 4.84 points for women. Gender did not significantly affect treatment outcomes. The baseline–third month change was 3.72 points in patients with a positive family history and 3.38 points in those with a negative history, and similar improvement was achieved in both groups over the six‐month period. Family history was also not decisive on treatment response (Table 2).

### 3.4 Clinical Response Categories

At the third month, the PRP group showed 4 no‐response, 7 partial, 7 good, and 12 very good responses; the ILTA group showed 5 no‐response, 8 partial, 7 good, and 10 very good responses. At six months, the very good response rate rose to 76.67% (23 patients) for PRP and 66.67% (20 patients) for ILTA. The number of non‐responders decreased to 2 in PRP and 3 in ILTA. The difference in the very good response rate at six months was 10.0 percentage points (95% CI −12.66 to 32.66; *p* = 0.390), which was not statistically significant (Table 4).

**TABLE 4:** Clinical response categories at 3 and 6 months.

Response category	3rd month PRP	3rd month ILTA	6th month PRP	6th month ILTA
No response	4 (13.33)	5 (16.67)	2 (6.67)	3 (10.00)
Partial response	7 (23.33)	8 (26.67)	2 (6.67)	3 (10.00)
Good response	7 (23.33)	7 (23.33)	3 (10.00)	4 (13.33)
Very good response	12 (40.00)	10 (33.33)	23 (76.67)	20 (66.67)
Total	30 (100.00)	30 (100.00)	30 (100.00)	30 (100.00)

Very good response (≥ 75%)	PRP	ILTA	Difference	95% CI	*p*
3rd month	12 (40.00)	10 (33.33)	6.67	−17.66 to 31.00	0.592
6th month	23 (76.67)	20 (66.67)	10.00	−12.66 to 32.66	0.390

*Note:* Values are *n* (%). Response categories: no response, < 25% SALT reduction; partial, 25%–49%; good, 50%–74%; very good, ≥ 75%. Difference in very good response rates tested with the chi‐square test; 95% CI by the Wald method. *p* < 0.05 was significant.

Abbreviations: CI, confidence interval; ILTA, intralesional triamcinolone acetonide; PRP, platelet‐rich plasma; SALT, Severity of Alopecia Tool.

### 3.5 Hair Pull Test Findings

The hair pull test was analyzed separately for each group. In the baseline–third month comparison, the test remained negative in 41 patients overall, and 10 patients changed from positive to negative (5 in the PRP group and 5 in the ILTA group). At six months, 13 patients transitioned from positive to negative (7 in PRP and 6 in ILTA), and 6 patients remained positive (3 in each group). The within‐group changes were statistically significant (*p* < 0.05), while the between‐group comparison of positive‐to‐negative conversion showed no significant difference (Fisher exact *p* = 1.000 at both the third and sixth months) (Table 5).

**TABLE 5:** Hair pull test conversion by treatment group.

Comparison	Group	Neg → Neg	Pos → Neg	Pos → Pos	*p* (within)^a^	*p* (between)^b^
Baseline–3rd month	PRP	20 (66.67)	5 (16.67)	5 (16.67)	0.063	1.000
	ILTA	21 (70.00)	5 (16.67)	4 (13.33)	0.063	
Baseline–6th month	PRP	20 (66.67)	7 (23.33)	3 (10.00)	0.016	1.000
	ILTA	21 (70.00)	6 (20.00)	3 (10.00)	0.031	
3rd–6th month	PRP	25 (83.33)	2 (6.67)	3 (10.00)	0.500	1.000
	ILTA	26 (86.67)	1 (3.33)	3 (10.00)	1.000	

*Note:* Values are *n* (%). A positive hair pull test was defined as more than six hairs extracted with gentle traction.

Abbreviations: ILTA, intralesional triamcinolone acetonide; Neg, negative; Pos, positive; PRP, platelet‐rich plasma.

^a^ Within‐group change between paired time points (exact McNemar test).

^b^ Between‐group difference in positive‐to‐negative conversion (Fisher exact test). *p* < 0.05 was significant.

### 3.6 Side Effect Profile and Safety Assessment

Only minor side effects such as mild pain, burning, ecchymosis, and tenderness were observed in both groups; all were Grade 1 and self‐limiting, and no serious side effects were reported. The most frequent event was injection‐site pain (21 patients in the PRP group and 19 in the ILTA group), followed by tenderness and transient erythema or edema, with comparable frequencies between the groups (all *p* > 0.05). Skin atrophy, telangiectasia, and hypopigmentation, which are associated with corticosteroid use in the literature, were not observed in either group during the follow‐up period. Because PRP is an autologous preparation, allergic and systemic reactions are unlikely, and none were observed in this study; however, given the limited sample size and the short follow‐up, these data should be interpreted as the absence of observed events rather than the absence of risk (Table 6).

**TABLE 6:** Treatment‐related adverse events during 6‐month follow‐up.

Adverse event	PRP (*n* = 30)	ILTA (*n* = 30)	Severity^a^	Median duration	*p*
Injection‐site pain	21 (70.00)	19 (63.33)	Grade 1	2 days	0.787
Burning sensation	9 (30.00)	7 (23.33)	Grade 1	1 day	0.774
Ecchymosis	7 (23.33)	6 (20.00)	Grade 1	7 days	1.000
Transient erythema/edema	11 (36.67)	9 (30.00)	Grade 1	2 days	0.787
Tenderness	14 (46.67)	12 (40.00)	Grade 1	3 days	0.794
Skin atrophy	0 (0.00)	0 (0.00)	—	—	1.000
Telangiectasia	0 (0.00)	0 (0.00)	—	—	1.000
Hypopigmentation	0 (0.00)	0 (0.00)	—	—	1.000
Local infection	0 (0.00)	0 (0.00)	—	—	1.000
Vasovagal reaction	1 (3.33)	0 (0.00)	Grade 1	< 1 h	1.000
Serious adverse event	0 (0.00)	0 (0.00)	—	—	1.000

*Note:* Values are *n* (%).

Abbreviations: ILTA, intralesional triamcinolone acetonide; PRP, platelet‐rich plasma.

^a^ Graded with the Common Terminology Criteria for Adverse Events version 5.0; Grade 1 denotes mild events requiring no intervention. Groups compared with the Fisher exact test; *p* < 0.05 was significant. All events were transient and self‐limiting. Skin atrophy, telangiectasia, and hypopigmentation were not observed in either group; given the sample size and follow‐up duration, the absence of an event reflects no observation within this study rather than absence of risk.

### 3.7 Relapse During Follow‐Up

Relapse during the six‐month follow‐up was infrequent in both groups, occurring in 1 of 30 patients (3.33%) in the PRP group and 2 of 30 patients (6.67%) in the ILTA group. The between‐group difference was −3.33 percentage points (95% CI −14.33 to 7.67; Fisher exact *p* = 1.000) and did not reach statistical significance. Given the small number of relapse events, this comparison should be interpreted with caution (Table 7).

**TABLE 7:** Relapse during 6‐month follow‐up.

Outcome	PRP (*n* = 30)	ILTA (*n* = 30)	Difference	95% CI	*p*
Relapse, *n* (%)	1 (3.33)	2 (6.67)	−3.33	−14.33 to 7.67	1.000
Within‐group rate, 95% CI^a^	0.08 to 17.22	0.81 to 22.07	—	—	—

*Note:* Values are *n* (%). Relapse was defined as a new alopecic patch or a ≥ 25% rise in SALT score above the post‐treatment nadir in a patient with prior response.

Abbreviations: CI, confidence interval; ILTA, intralesional triamcinolone acetonide; PRP, platelet‐rich plasma; SALT, Severity of Alopecia Tool.

^a^ Clopper–Pearson exact method; between‐group difference by the Wald method and the Fisher exact test. *p* < 0.05 was significant.

### 3.8 Clinical Presentation

**FIGURE 2:**
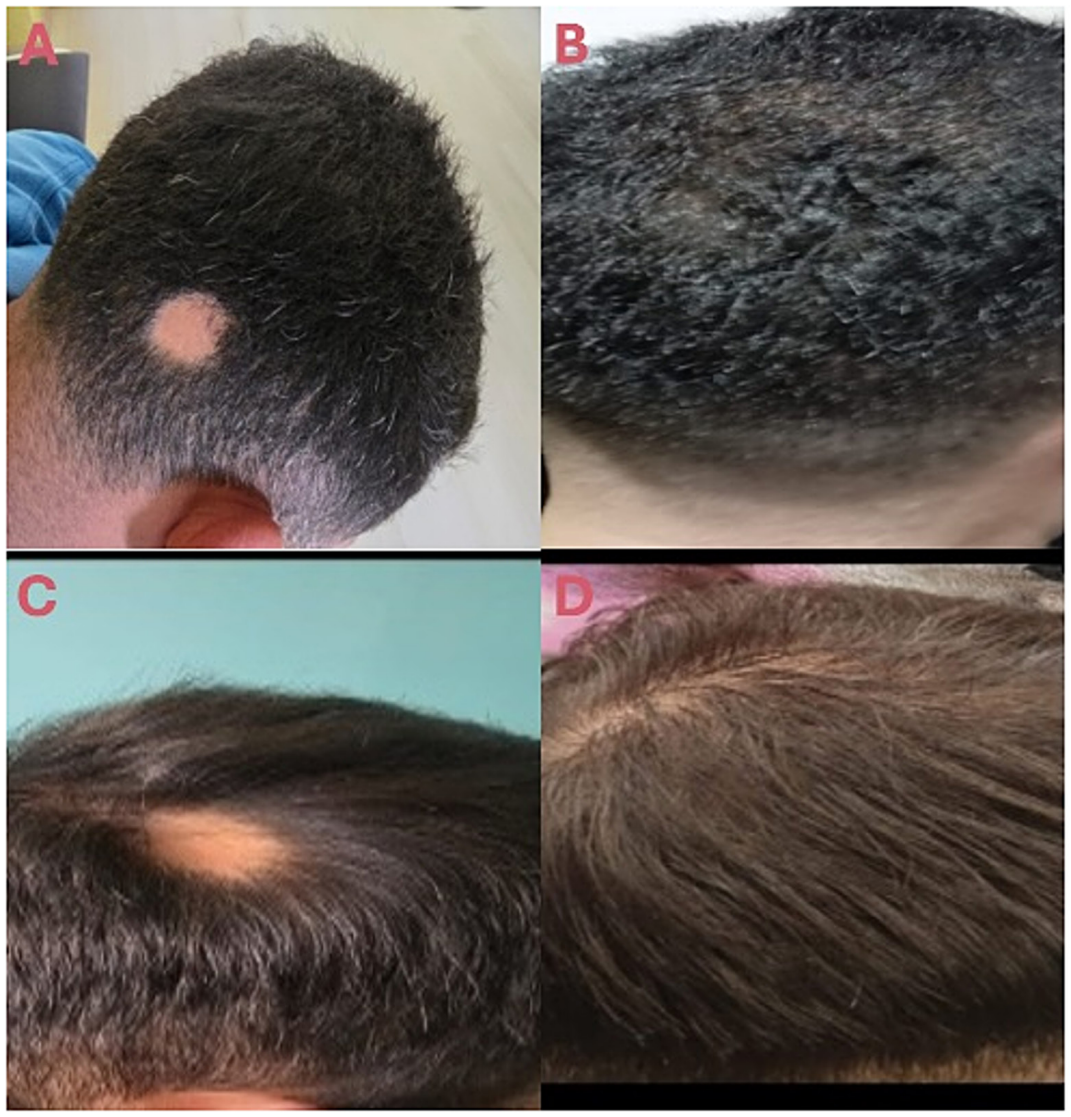
Representative clinical response of patients with alopecia areata treated with platelet‐rich plasma (PRP). (A) Alopecia areata patch in the first patient before PRP treatment; (B) near‐complete hair regrowth in the same patient after three sessions of PRP treatment; (C) alopecia areata patch in the second patient before PRP treatment; (D) near‐complete hair regrowth with increased pigmentation in the patch of the same patient after three sessions of PRP treatment. These photographs are provided for illustrative purposes only and were not standardized for camera angle, lighting, hair length, or scalp exposure. Written informed consent for publication of clinical images was obtained from all patients shown.

**FIGURE 3:**
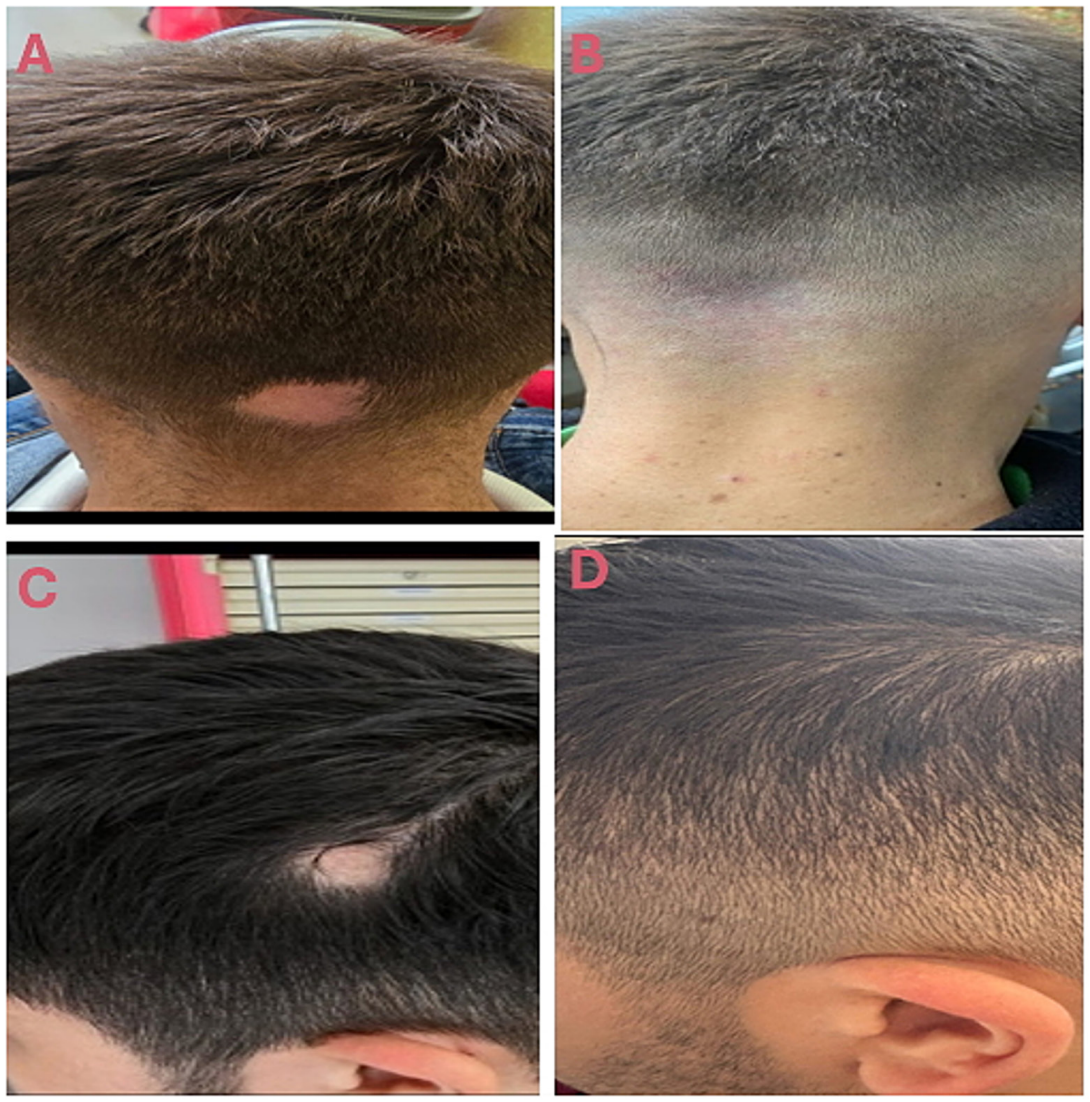
Representative clinical response of patients with alopecia areata treated with intralesional triamcinolone acetonide (ILTA). (A) Alopecia areata patch in the first patient before ILTA treatment; (B) marked hair regrowth and resolution of the patch in the same patient after three sessions of ILTA treatment; (C) alopecia areata patch in the second patient before ILTA treatment; (D) marked hair regrowth and resolution of the patch in the same patient after three sessions of ILTA treatment. These photographs are provided for illustrative purposes only and were not standardized for camera angle, lighting, hair length, or scalp exposure. Written informed consent for publication of clinical images was obtained from all patients shown.

Near‐complete hair regrowth was observed after three sessions in patients who received PRP treatment, with increased pigmentation noted in some cases (Figure 2). A similar level of clinical improvement was observed in the ILTA group, where the alopecia areata patches closed substantially (Figure 3). Clinical responses in both treatment groups were consistent with the statistical analyses.

## 4 Discussion

This study was conducted to compare the efficacy and safety of platelet‐rich plasma (PRP) and intralesional triamcinolone acetonide (ILTA) in patients with mild patch‐type alopecia areata. The potential influence of prognostic factors—including age, gender, family history, and disease duration—on treatment response was also examined. The findings demonstrated that both treatment modalities produced significant clinical improvement, and no statistically significant difference was detected between the two groups. These results suggest that PRP produces improvement comparable to that of ILTA and may offer patients a steroid‐free treatment alternative.

In our study, the baseline SALT score was 6.03 ± 0.89 in the PRP group and 6.07 ± 0.78 in the ILTA group; all patients were classified as S1 (mild severity). A significant reduction in SALT score was observed after both treatment approaches. In the PRP group, the SALT score decreased from 6.03 at baseline to 2.53 at 3 months and 0.93 at 6 months; in the ILTA group, it decreased from 6.07 at baseline to 2.60 at 3 months and 1.27 at 6 months. The between‐group difference in SALT change at 6 months was small and not statistically significant (0.30; 95% CI −0.77 to 1.37; *p* = 0.576), and the confidence interval included zero, representing insufficient evidence for a clinically meaningful treatment advantage.

Similar results have been reported in the literature. Fawzy et al. [18] observed significant improvement in SALT scores following both PRP and ILTA treatment but found no significant difference between the groups. Similarly, Balakrishnan et al. [19] reported significant clinical improvement with both modalities without demonstrating superiority of either treatment. However, findings in the literature remain inconsistent. Kapoor et al. [8] reported better outcomes with ILTA, whereas Trink et al. [20] found PRP to be more effective in promoting hair growth. These discrepancies may be attributed to variations in study design, PRP preparation protocols, platelet concentrations, injection techniques, and patient characteristics, including disease severity and duration. Our cohort consisted only of patients with mild, recent‐onset disease, and this is likely to have contributed to the strong response seen in both arms. The lack of a placebo or untreated control group should be considered when interpreting the detected improvement.

At the sixth month, a very good response (≥ 75% improvement) was achieved in 76.67% of the PRP group and 66.67% of the ILTA group. Non‐response rates remained low (PRP 6.66%, ILTA 10%). It has been consistently reported in the literature that both treatments provide significant improvement, with no clear superiority of one over the other [8, 10, 18, 19, 20, 21, 22]. El Taieb et al. [23] observed complete clinical response at 3 months in 70% of PRP patients. Trink et al. [20] reported 60% remission in PRP and 27% remission in ILTA at 9‐month follow‐up. Albalat and Ebrahim [21] achieved a 65% improvement in ILTA and a 72.5% improvement in PRP, with no significant difference between the groups. Our findings are consistent with these data. The difference in the very good response rate between the two groups at six months was 10.0 percentage points (95% CI −12.66 to 32.66; *p* = 0.390) and did not reach statistical significance.

An advantage of PRP is its autologous origin: because it is prepared from the patient's own blood, the growth factors and cytokines released from platelet α‐granules can stimulate follicular repair and cellular proliferation while carrying little risk of allergic or systemic reactions [13, 18]. This biological mechanism is the rationale most often cited for using PRP in alopecia areata.

In terms of safety, only mild pain, burning, ecchymosis, and tenderness were observed in both groups; no serious side effects were reported. Skin atrophy, telangiectasia, and hypopigmentation have been reported in the literature with long‐term use of ILTA [10]. None of these steroid‐related changes were seen in our patients during the 6‐month follow‐up, which may be related to the use of 1‐cm injection intervals and the avoidance of superficial application; however, the study was not powered to detect uncommon adverse events, so their absence here should not be read as proof that the risk is zero. PRP, as a simple and easily applicable preparation, has been associated with a favorable safety profile [23]. Singh [24] reported 95% remission at 6 months of follow‐up. Balakrishnan et al. [19] reported severe pain in three patients in the PRP group, while no serious side effects were detected in the ILTA group. Because PRP avoids steroid‐related side effects, it may be a useful option for patients who require prolonged therapy.

The potential influence of prognostic factors (gender, family history, age, and disease duration) on treatment response was analyzed; no statistically significant differences were identified. No difference was observed in SALT change between male (*n* = 41) and female (*n* = 19) patients (B–3rd month: 3.37 ± 2.07 vs. 3.74 ± 2.13, *p* = 0.951; B–6th month: 5.00 ± 2.01 vs. 4.84 ± 2.19, *p* = 0.576). Similarly, no difference was found between patients with negative (*n* = 42) and positive (*n* = 18) family history (B–3rd month: 3.38 ± 2.11 vs. 3.72 ± 2.05, *p* = 0.565; B–6th month: 4.95 ± 2.04 vs. 4.94 ± 2.15, *p* = 0.989). These findings suggest that these prognostic factors do not influence the choice of treatment modality.

Kumar et al. [25] reported a male predominance (80%) among alopecia areata patients. A similar male predominance (68.33%) was observed in our study, although no difference was detected in treatment response according to gender. Fawzy et al. [18] reported that family history and comorbidities did not affect treatment response. In contrast, a retrospective analysis indicated that patients with a family history of alopecia areata had inferior treatment outcomes [26]. In our study, although 18 patients had a positive family history, no significant difference was detected in treatment response. This suggests that family history may influence disease course but not therapeutic response.

The mean age was 27.97 ± 7.36 years, which is consistent with the literature. Kumar et al. [25] reported a median disease duration of 3.3 ± 1.8 months and a 70% very good response rate. Shumez et al. [27] observed a disease duration of 1–6 months in 74.32% of patients and a complete response after three sessions of PRP. In our study, the mean disease duration was 5 ± 7.11 months, and the complete response rate was higher in patients with disease duration of less than 12 months. The relatively young mean age, short disease duration, and predominance of S1 disease (< 25% scalp involvement) may collectively explain the high treatment response rates observed in our cohort.

The hair pull test serves as an indicator of disease activity in alopecia areata. At baseline, 19 patients (31.7%) had a positive hair pull test. By the third month, a conversion from positive to negative was observed in 10 patients (*χ* ^2^ = 22.85; *p* = 0.001). By the sixth month, 13 patients had converted from positive to negative, while only six patients (10%) remained positive (*χ* ^2^ = 14.39; *p* = 0.001). When examined by group, the change from positive to negative was similar in the PRP and ILTA arms (Fisher exact *p* = 1.000 at both the third and sixth months), indicating that neither treatment was superior in this respect. The parallel improvement in SALT scores and hair pull test findings supports the efficacy of both treatments, as the positivity rate decreased from 31.7% to 10%.

During the 6‐month follow‐up, relapse occurred in 3 of 60 patients (5.0%): 1 of 30 patients (3.33%) in the PRP group and 2 of 30 patients (6.66%) in the ILTA group. The between‐group difference was not significant (−3.33 percentage points; 95% CI −14.33 to 7.67; Fisher exact *p* = 1.000), and given the small number of events, this comparison should be read with caution. Albalat and Ebrahim [21] reported a relapse rate of 25% in ILTA patients and only 5% in PRP patients. Singh [24] reported that 5% of participants experienced relapse, while 95% achieved remission. Trink et al. [20] reported that PRP treatment achieved 60% remission, whereas ILTA treatment resulted in only 27% remission during the 9‐month follow‐up period. Kapoor et al. [8] reported one recurrence in the ILTA group and two recurrences in the PRP group. The low relapse rate in our study is in line with the literature but should be interpreted in the context of the mild, recent‐onset disease in our cohort, since spontaneous remission is common during the first year of alopecia areata. Taken together, the hair pull test findings and the low relapse rate suggest that both PRP and ILTA provide durable therapeutic benefit in patients with mild patch‐type alopecia areata.

This study has some limitations. It was guided at a single center with a relatively small sample size and included only patients with mild patch‐type alopecia areata. In addition, the follow‐up period was limited to 6 months. Although the present findings support the efficacy and safety of PRP in mild patch‐type alopecia areata, larger randomized controlled studies with longer follow‐up are required to validate the findings of the present study results and to enhance the generalizability of the results to broader patient populations.

## 5 Conclusion

In adults with mild, untreated patch‐type alopecia areata, PRP produced SALT score improvements comparable to those of intralesional triamcinolone acetonide over a 6‐month period, with only mild adverse events observed in both groups. The demographic and clinical characteristics of the patients did not significantly affect treatment response. As an autologous biological product derived from the patient's own blood, PRP may represent a valuable treatment alternative for patients who prefer to avoid the potential adverse effects associated with corticosteroid therapy.

## Author Contributions

Conceptualization: R.Ü., İ.Y.O., H.U.Ö.; Data curation: R.Ü., İ.Y.O., H.U.Ö.; Formal analysis: R.Ü., İ.Y.O., H.U.Ö.; Funding acquisition: R.Ü., İ.Y.O.; Investigation: R.Ü., İ.Y.O., H.U.Ö.; Methodology: R.Ü., İ.Y.O., H.U.Ö.; Project administration: R.Ü., İ.Y.O.; Resources: R.Ü., İ.Y.O., H.U.Ö.; Software: R.Ü.; Supervision: İ.Y.O., H.U.Ö.; Validation: R.Ü., İ.Y.O., H.U.Ö.; Visualization: R.Ü., İ.Y.O., H.U.Ö.; Writing – original draft: R.Ü., İ.Y.O., H.U.Ö.; Writing – review and editing: R.Ü., İ.Y.O., H.U.Ö.

## Funding

This study was supported by the Coordinatorship of Scientific Research Projects of Van Yüzüncü Yıl University (Project No. TTU‐2024‐11063).

## Disclosure

No artificial intelligence–assisted tools were used to generate scientific content, analyze data, or interpret results in this study. AI‐based tools were not used in the preparation of the manuscript.

## Ethics Statement

This prospective study was approved by the Clinical Research Ethics Committee of Van Yüzüncü Yıl University (Approval No: 11063, Date: 28.01.2024).

## Consent

Written informed consent was obtained from all participants prior to enrollment.

## Conflicts of Interest

The authors declare no conflicts of interest.

## Data Availability

The data that support the findings of this study are available on request from the corresponding author. The data are not publicly available due to privacy or ethical restrictions.

## References

[1] C. H. Pratt , L. E. King, Jr. , A. G. Messenger , A. M. Christiano , and J. P. Sundberg , “Alopecia Areata,” Nature Reviews Disease Primers 3 (2017): 17011, 10.1038/nrdp.2017.11. PMC5573125 28300084

[2] F. Spano and J. C. Donovan , “Alopecia Areata: Part 2: Treatment,” Canadian Family Physician 61, no. 9 (2015): 757–761. 26371098 PMC4569105

[3] T. Simakou , J. P. Butcher , S. Reid , and F. L. Henriquez , “Alopecia Areata: A Multifactorial Autoimmune Condition,” Journal of Autoimmunity 98 (2019): 74–85, 10.1016/j.jaut.2018.12.001. 30558963

[4] A. Sterkens , J. Lambert , and A. Bervoets , “Alopecia Areata: A Review on Diagnosis, Immunological Etiopathogenesis and Treatment Options,” Clinical and Experimental Medicine 21, no. 2 (2021): 215–230, 10.1007/s10238-020-00673-w. 33386567

[5] D. Tzur Bitan , D. Berzin , K. Kridin , and A. Cohen , “The Association Between Alopecia Areata and Anxiety, Depression, Schizophrenia, and Bipolar Disorder: A Population‐Based Study,” Archives of Dermatological Research 314, no. 5 (2022): 463–468, 10.1007/s00403-021-02247-6. 34089375

[6] N. Yu and Y. Guo , “Association Between Alopecia Areata, Anxiety, and Depression: Insights From a Bidirectional Two‐Sample Mendelian Randomization Study,” Journal of Affective Disorders 350 (2024): 328–331, 10.1016/j.jad.2024.01.152. 38242214

[7] C. Zhou , X. Li , C. Wang , and J. Zhang , “Alopecia Areata: An Update on Etiopathogenesis, Diagnosis, and Management,” Clinical Reviews in Allergy and Immunology 61, no. 3 (2021): 403–423, 10.1007/s12016-021-08883-0. 34403083

[8] P. Kapoor , S. Kumar , B. K. Brar , N. Kukar , H. Arora , and S. K. Brar , “Comparative Evaluation of Therapeutic Efficacy of Intralesional Injection of Triamcinolone Acetonide Versus Intralesional Autologous Platelet‐Rich Plasma Injection in Alopecia Areata,” Journal of Cutaneous and Aesthetic Surgery 13, no. 2 (2020): 103–111, 10.4103/JCAS.JCAS_16_19. 32792771 PMC7394112

[9] E. H. C. Wang , B. N. Sallee , C. I. Tejeda , and A. M. Christiano , “JAK Inhibitors for Treatment of Alopecia Areata,” Journal of Investigative Dermatology 138, no. 9 (2018): 1911–1916, 10.1016/j.jid.2018.05.027. 30057345 PMC6475885

[10] P. Hegde , V. Relhan , B. Sahoo , and V. K. Garg , “A Randomized, Placebo and Active Controlled, Split Scalp Study to Evaluate the Efficacy of Platelet‐Rich Plasma in Patchy Alopecia Areata of the Scalp,” Dermatologic Therapy 33, no. 6 (2020): e14388, 10.1111/dth.14388. 33034942

[11] D. A. Lintzeri , A. Constantinou , K. Hillmann , K. Ghoreschi , A. Vogt , and U. Blume‐Peytavi , “Alopecia Areata – Current Understanding and Management,” Journal der Deutschen Dermatologischen Gesellschaft 20, no. 1 (2022): 59–90, 10.1111/ddg.14689. 35040577

[12] R. Alves and R. Grimalt , “A Review of Platelet‐Rich Plasma: History, Biology, Mechanism of Action, and Classification,” Skin Appendage Disorders 4, no. 1 (2018): 18–24, 10.1159/000477353. 29457008 PMC5806188

[13] J. Stevens and S. Khetarpal , “Platelet‐Rich Plasma for Androgenetic Alopecia: A Review of the Literature and Proposed Treatment Protocol,” International Journal of Women's Dermatology 5, no. 1 (2018): 46–51, 10.1016/j.ijwd.2018.08.004. PMC6374694 30809579

[14] H. M. Almohanna , A. A. Ahmed , J. W. Griggs , and A. Tosti , “Platelet‐Rich Plasma in the Treatment of Alopecia Areata: A Review,” Journal of Investigative Dermatology. Symposium Proceedings 20, no. 1 (2020): S45–S49, 10.1016/j.jisp.2020.05.002. 33099384

[15] National Cancer Institute, Division of Cancer Treatment and Diagnosis , “Common Terminology Criteria for Adverse Events (CTCAE) Version 5.0,” U.S. Department of Health and Human Services, (2017).

[16] J. Magalon , A. L. Chateau , B. Bertrand , et al., “DEPA Classification: A Proposal for Standardising PRP Use and a Retrospective Application of Available Devices,” BMJ Open Sport & Exercise Medicine 2, no. 1 (2016): e000060, 10.1136/bmjsem-2015-000060. PMC5117023 27900152

[17] A. J. Vickers and D. G. Altman , “Statistics Notes: Analysing Controlled Trials With Baseline and Follow Up Measurements,” BMJ 323, no. 7321 (2001): 1123–1124, 10.1136/bmj.323.7321.1123. 11701584 PMC1121605

[18] M. M. Fawzy , R. Abdel Hay , F. N. Mohammed , K. S. Sayed , M. E. D. Ghanem , and M. Ezzat , “Trichoscopy as an Evaluation Method for Alopecia Areata Treatment: A Comparative Study,” Journal of Cosmetic Dermatology 20, no. 6 (2021): 1827–1836, 10.1111/jocd.13739. 32991045

[19] A. Balakrishnan , B. Joy , A. Thyvalappil , P. Mathew , A. Sreenivasan , and R. Sridharan , “A Comparative Study of Therapeutic Response to Intralesional Injections of Platelet‐Rich Plasma Versus Triamcinolone Acetonide in Alopecia Areata,” Indian Dermatology Online Journal 11, no. 6 (2020): 920–924, 10.4103/idoj.IDOJ_6_20. 33344340 PMC7734998

[20] A. Trink , E. Sorbellini , P. Bezzola , et al., “A Randomized, Double‐Blind, Placebo‐ and Active‐Controlled, Half‐Head Study to Evaluate the Effects of Platelet‐Rich Plasma on Alopecia Areata,” British Journal of Dermatology 169, no. 3 (2013): 690–694, 10.1111/bjd.12397. 23607773

[21] W. Albalat and H. M. Ebrahim , “Evaluation of Platelet‐Rich Plasma vs Intralesional Steroid in Treatment of Alopecia Areata,” Journal of Cosmetic Dermatology 18, no. 5 (2019): 1456–1462, 10.1111/jocd.12858. 31074201

[22] A. Paichitrojjana and A. Paichitrojjana , “Platelet Rich Plasma and Its Use in Hair Regrowth: A Review,” Drug Design, Development and Therapy 16 (2022): 635–645, 10.2147/DDDT.S356858. 35300222 PMC8922312

[23] M. A. El Taieb , H. Ibrahim , E. A. Nada , and M. Seif Al‐Din , “Platelets Rich Plasma Versus Minoxidil 5% in Treatment of Alopecia Areata: A Trichoscopic Evaluation,” Dermatologic Therapy 30, no. 1 (2017), 10.1111/dth.12437. 27791311

[24] S. Singh , “Role of Platelet‐Rich Plasma in Chronic Alopecia Areata: Our Centre Experience,” Indian Journal of Plastic Surgery 48, no. 1 (2015): 57–59, 10.4103/0970-0358.155271. 25991888 PMC4413492

[25] A. Kumar , R. P. Sharma , S. Bali , and P. Arya , “Role of Platelet Rich Plasma Therapy in Alopecia Areata: A Prospective Study,” International Journal of Contemporary Medical Research 3, no. 8 (2016): 2499–2502.

[26] S. Wang , R. Ratnaparkhi , M. Piliang , and W. F. Bergfeld , “Role of Family History in Patchy Alopecia Areata,” Dermatology Online Journal 24, no. 10 (2018): 13030/qt0n19r7ps. 30677822

[27] H. Shumez , P. V. S. Prasad , P. K. Kaviarasan , and R. Deepika , “Intralesional Platelet Rich Plasma vs Intralesional Triamcinolone in the Treatment of Alopecia Areata: A Comparative Study,” International Journal of Medical Research & Health Sciences 4, no. 1 (2015): 118–122, 10.5958/2319-5886.2015.00019.3.

